# Primary headache types in adult epilepsy patients

**DOI:** 10.1186/s40001-023-01023-8

**Published:** 2023-01-27

**Authors:** Katharina Schiller, Markus Rauchenzauner, Tamir Avidgor, Sana Hannan, Carlo Lorenzen, Manuela Kaml, Gerald Walser, Iris Unterberger, Vera Filippi, Gregor Broessner, Gerhard Luef

**Affiliations:** 1 Department of Pediatrics and Neonatology, Hospital Ostallgaeu, Kaufbeuren, Germany; 2 Analytical Neurophysiology Lab, Montreal Neurological Institute and Hospital, McGill University, Montreal, Canada; 3 Department of Pediatrics, Medical University Innsbruck, Innsbruck, Austria; 4 Department of Neurology, Epilepsy Unit, Medical University Innsbruck, Anichstrasse 35, Innsbruck, 6020 Austria; 5 Department of Neurology, Headache Outpatient Clinic, Medical University Innsbruck, Anichstrasse 35, Innsbruck, 6020 Austria

**Keywords:** Headache, Epilepsy, AED, Migraine, Tension-type headache

## Abstract

**Background:**

Headache is among the most common comorbidities in epilepsy. This study examined the distribution of different primary headache disorders in a large cohort of patients with diagnosed epilepsy. Headache types were analysed with regard to gender, type of epilepsy and antiepileptic drugs (AEDs).

**Methods:**

In this prospective single-centre study, 500 patients with epilepsy (250 female, mean age: 45.52 ± 17.26 years) were evaluated with regards to primary headache types using a validated German headache questionnaire categorizing for migraine (MIG), tension-type headache (TTH) or trigeminal autonomic cephalalgias (TAC), their combinations and unclassifiable headache. Data regarding type of epilepsy, seizure-associated headache, AED treatment and seizure freedom were collected.

**Results:**

Of 500 patients with epilepsy, 163 (32.6%) patients (108 female and 55 male) reported suffering from headaches at least 1 day per month. MIG (without aura, with aura) and TTH were the most frequent headache type (MIG 33.1%, TTH 33.1%). Female epilepsy patients reported headaches significantly more often than male patients (*x*^2^ = 8.20, *p* = 0.0042). In contrast, the type of epilepsy did not significantly affect headache distribution. Of 163 patients with headache, 66 (40.5%) patients reported seizure-associated headache and AEDs were used by 157 patients. Of importance, patients with AED monotherapy suffered from MIG less often when compared to patients on polytherapy (*x*^2^ = 4.79, *p* = 0.028).

**Conclusion:**

MIG and TTH are the most common headache types in epilepsy patients and headache is more frequent among female epilepsy patients. Monotherapy in AEDs might have a beneficial effect on the frequency of headache compared to polytherapy.

## Introduction

Epilepsy is known to be associated with a variety of comorbidities with headache being one of the most frequently reported [1]. This may be explained by the shared common pathophysiology between epilepsy and headache, especially migraine, and the influence of a genetic disposition [2, 3].

Headache can be divided into primary and secondary headaches: whereas primary headaches have no apparent underlying cause, secondary headaches are defined as being caused by another disorder such as neck trauma, vascular disorders or cerebral hemorrhage [4]. According to the latest edition of the International Classification of Headache Disorders (ICHD-3), primary headache types are migraine (MIG), tension-type headaches (TTC), trigeminal autonomic cephalgia (TAC) and neuralgias [5].

MIG is typically characterised by unilateral, pulsating headache of moderate or severe intensity, aggravated by physical activity, associated with nausea and sensitivity to light or sound and can occur with or without aura. The association between epilepsy and migraine is well established, but the rate of prevalence varies widely across studies [6–8]. In a meta-analysis from Duko et al., 26% of epilepsy patients reported MIG with aura, whereas MIG without aura was prevalent in 10.4% [9]. To date, there are two antiepileptic drugs (AEDs), topiramate and valproic acid, which are approved for both MIG prophylaxis and treatment of epilepsy [10–17]. Topiramate was also shown to reduce TTH [18].

TTH is bilaterally located, of a pressing or tightening quality (non-pulsating), with mild to moderate intensity, not aggravated by physical activity and not accompanied by other symptoms [5]. TTH is quite common in the general population [19] and among epilepsy patients; the frequency of 26.2% is comparable to the prevalence rate of MIG [9]. In general, women are more often diagnosed with MIG or TTH than men [9, 19].

TAC such as cluster headache is described as severe or very severe unilateral orbital, supraorbital and/or temporal pain lasting 15–180 min with ipsilateral conjunctival injection or lacrimation, nasal congestion, eyelid oedema, forehead and facial sweating and miosis, or a sense of restlessness [5]. Cluster headache is a severe and rare headache type and affects around 0.1% of the population with a predominance in men [20]. Data on cluster headache in the context of epilepsy are sparse [21].

The aim of our study was to investigate the distribution of the three different primary headache types, MIG, TTH and TAC, and potential influencing factors such as age or type of epilepsy in a population of patients with epilepsy. Epilepsy patients were sex-matched (250 female and 250 male participants) to study gender differences. Furthermore, the influence of different AEDs on headache as well as the relation between headache and seizure freedom are evaluated.

## Methods

In this prospective single-centre study, patients with epilepsy in the seizure outpatient clinic of the University Hospital of Innsbruck were evaluated. Informed consent was obtained from all patients in accordance with the Declaration of Helsinki and the study was approved by the local ethics committee.

The study population consisted of a large cohort of *n* = 500 sex-matched adult patients (250 female) with diagnosed epilepsy based on the ILAE classification [22]. Patients had to fulfill the criteria of a documented diagnosis of epilepsy, age of 18 years or older, and must have been able to fill out the questionnaire autonomously. The patients were separated into two groups: group 1 with headache and group 2 without headache. Patients were asked during their outpatient visit if they had a headache at least 1 day per month. If they reported having headaches one or more days per month, they were handed out the headache questionnaire with additional items about sociodemographic/epilepsy data and allocated to group 1. If they declined to have a headache at least 1 day per month, they did not fill out the headache questionnaire and continued with the questionnaire about the sociodemographic/epilepsy data and were allocated to group 2.

### Headache questionnaire

Headache was assessed using a validated headache questionnaire for screening for MIG, TTH TAC [23]. The questionnaire was based on the second version of the classification criteria of the International Headache Society (ICHD-3) [24] and consists of seven items for MIG plus two items to separate between MIG with aura and without aura, seven items for TTH and six items for TAC. The questions were to be answered with “yes” or “no”. After each block for MIG, TTH and TAC, the total amount of days per month for this block was registered to distinguish between chronic headache type (15 days per month or more) or episodic headache type (14 days and less) or episodic versus chronic cluster headache, respectively.

The algorithm allowed the following outcome results: MIG with and without aura, TTH, TAC, a combination of MIG and TTH (MIG + TTH), a combination of TTH and TAC (TTH + TAC), a combination of MIG and TAC (MIG + TAC) and “non-classifiable” headache (if all the criteria of the ICHD-3 for one headache type were not fulfilled).

### Sociodemographic/epilepsy data

We added additional items to assess data regarding epilepsy and medication. Sociodemographic data consisted of age, sex, date of birth and data regarding epilepsy comprised type of epilepsy (focal, generalised, unclassifiable), onset year of epilepsy, current medication for epilepsy (AEDs) and seizure freedom in the last 12 months (yes/no).

For the patients reporting headaches at least 1 day per month, further standardised items were added. The total number of days with headache per month, headache together with a seizure (before/during/after), difference in headache type during and outside of seizures (yes/no) and improvement of headache since the start of AEDs (yes/no) were evaluated.

### Statistics

Data are presented as absolute numbers and percentages in parentheses where appropriate. Normal distribution of the data was tested using the Kolmogorov–Smirnov test. Contingency tables and Pearson’s chi-squared test (*x*^2^-test) and Fisher’s exact test were applied. All analyses were performed two-tailed with p-values ≤ 0.05 indicating statistical significance. Statistical analyses were performed using the Statistical Package for Social Sciences for Windows (SPSS Inc. Chicago, Illinois, version 27.0).

## Results

500 sex-matched patients with diagnosed epilepsy participated in our study. Baseline clinical characteristics of all patients and patients with headache at least 1 day per month are presented in Table 1.

**Table 1:** Baseline clinical characteristics and types of epilepsy

	All patients with epilepsy	Epilepsy patients with headache
*n* =	500	163
Age, y (mean ± SD)	45.5 ± 17.3	42.8 ± 14.9
Sex, female/male	250/250	108/55
Age onset epilepsy, y (mean ± SD)	27.2 ± 20.6	24.6 ± 17.6
Duration epilepsy, y (mean ± SD)	18.3 ± 15.1	18.3 ± 15.1
Types of epilepsy, n (%)		
Focal	366 (73.2)	122 (74.8)
Generalised	118 (23.6)	37 (22.7)
Unclassifiable	16 (3.2)	4 (2.5)

### Distribution of headache

Overall, out of 500 epilepsy patients, 163 patients (32.6%) reported suffering from headache at least 1 day per month.

**Fig. 1:**
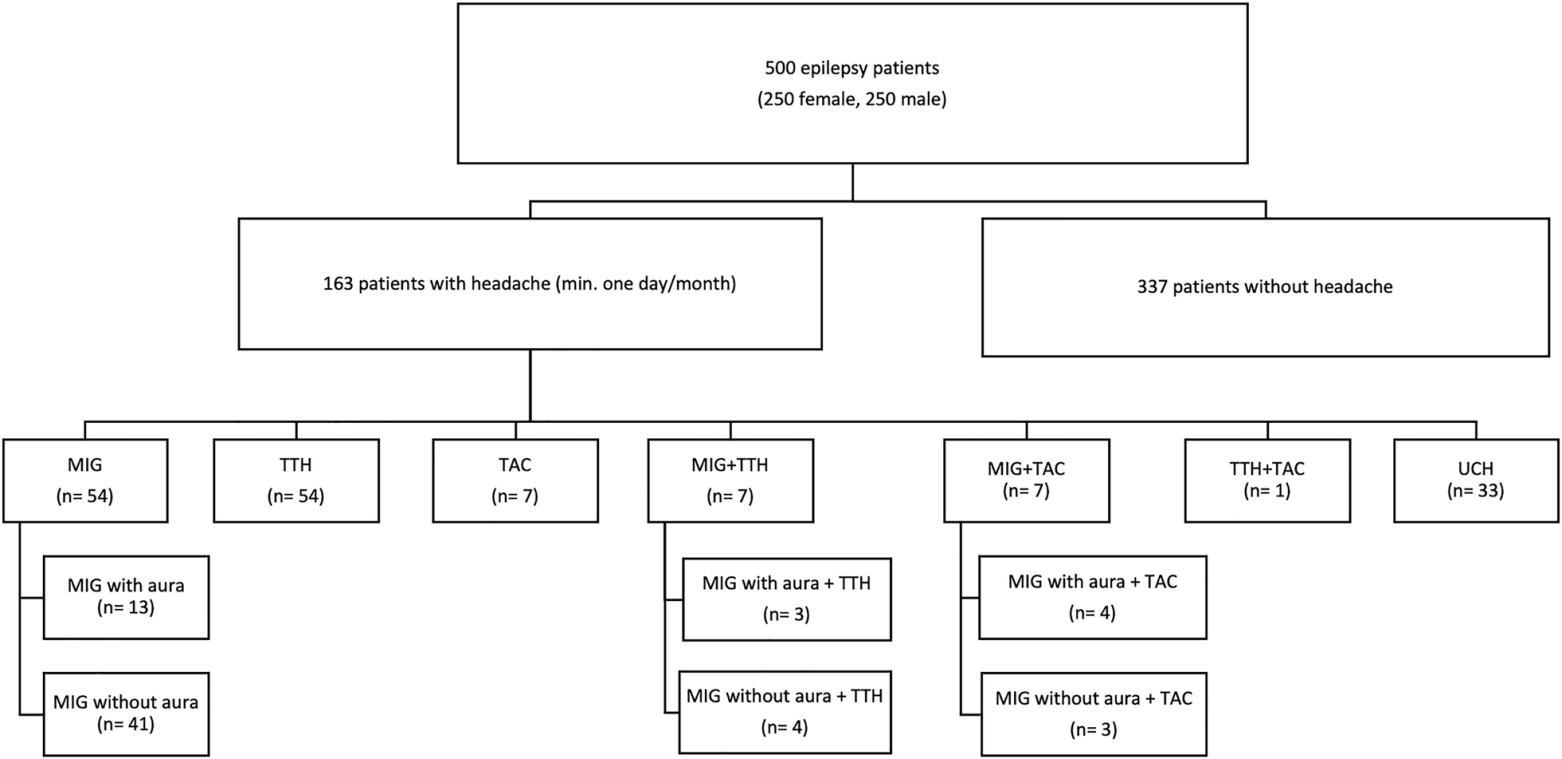
Flowchart presenting headache distribution. *MIG* migraine, *TTH* tension-type headache, *TAC* trigeminal autonomic cephalgia, *UCH* unclassifiable headache

In the 163 patients with headache, all 3 primary headache types and their combinations were reported by patients with epilepsy *(*Fig. 1). MIG was the most frequent form reported by 54 (33.1%), of which 41 had MIG without aura and 13 MIG with aura. This was followed by TTH in 54 (33.1%) patients and TAC with 7 (4.3%) patients. In 33 (20.2%) patients, answers did not meet the full criteria for one of the headache types (non-classifiable headache, NCH).

Altogether, headache was classified as chronic in 15 (9.2%) patients. 7 (4.3%) patients reported chronic MIG (without aura), 5 (3.1%) patients had chronic TTH and 3 (1.8%) patients described chronic TAC.

### Gender distribution

**Fig. 2:**
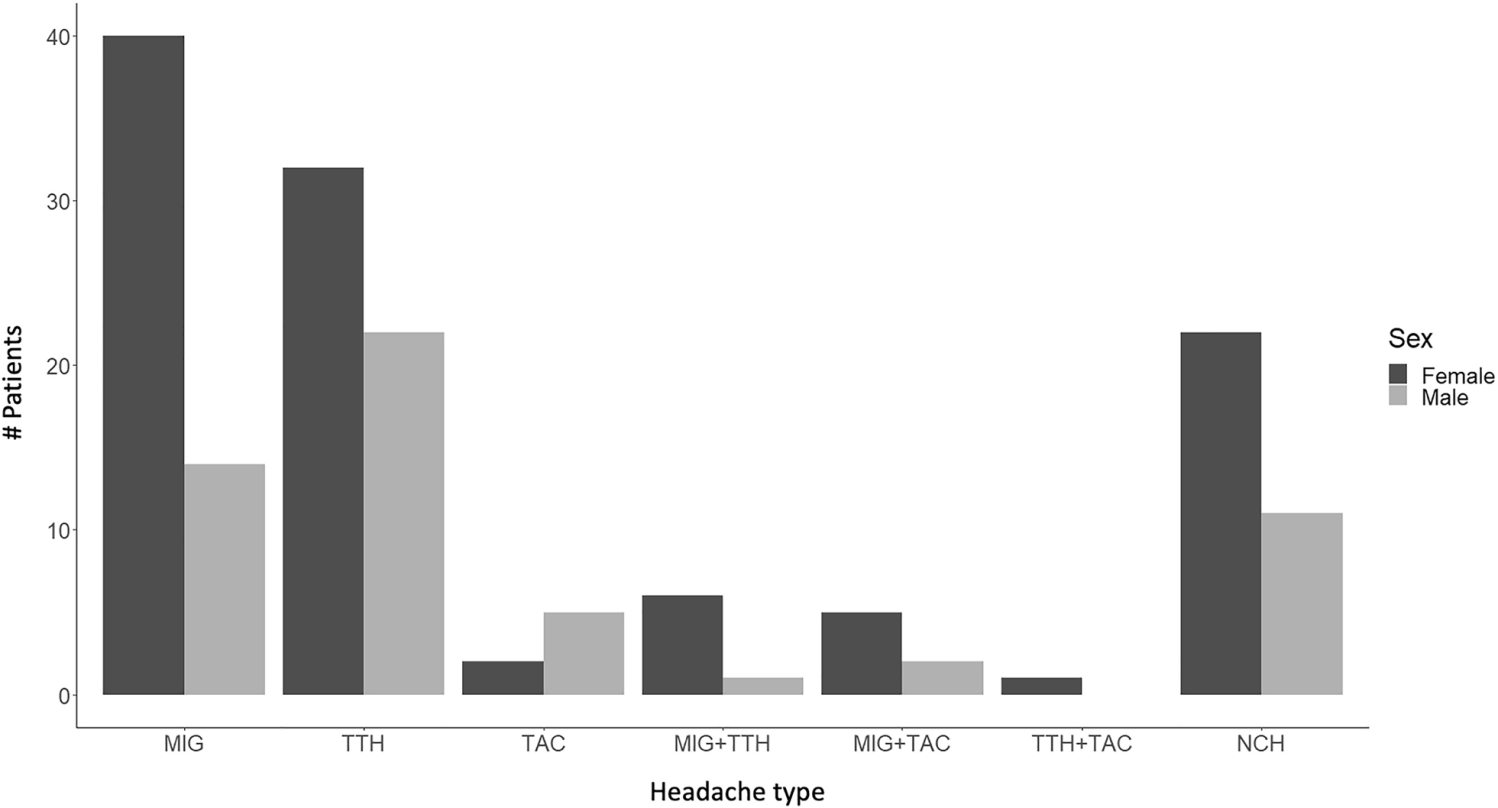
Different headache types for female (*n* = 108) and male (*n* = 55) epilepsy patients

Of 163 epilepsy patients with headache, more female patients (*n* = 108) had headache than male patients (*n* = 55) (Fig. 2). This difference was statistically significant (*x*^2^ = 8.20, *p* = 0.0042). Of 108 female patients with headache, 40 (37.0%) had MIG, of which 29 patients had MIG without aura and 11 had MIG with aura. TTH was found in 32 (29.6%) patients. TAC was reported by two (1.9%) patients. A combination of MIG + TTH was described by six (5.6%) patients (three without aura, three with aura) and a combination of MIG + TAC by five (4.6%) patients (three without aura, two with aura). One (0.9%) patient had a combination of TTH and TAC. Twenty-two (20.4%) patients showed NCH.

Of 55 male patients with headache, 14 (25.5%) of our patients with headache had MIG, whereof 12 (21.8%) patients had MIG without aura and 2 (3.6%) had MIG with aura. TTH was reported by 22 (40.0%), whereas TAC was described by 5 (9.1%) patients. A combination of MIG (without aura) + TTH was reported by one patient (1.8%) and two (3.6%) patients had a combination of MIG (with aura) + TAC. Eleven (20.0%) patients had NCH.

The distribution of the headache types was not significantly different between female and male patients (all *p* > 0.05).

Two women had chronic MIG, three men chronic MIG, two women chronic MIG + TAC, three women reported chronic MIG + TTH, one man chronic MIG + TTH, one woman and one man chronic TAC, two women chronic TTH, one man chronic TTH and one woman chronic TTH + TAC.

### Headache in relation to the type of epilepsy

In the study population of 500 patients with epilepsy, 366 patients had focal epilepsy, 118 patients had generalised epilepsy and 16 had unclassifiable epilepsy. 122 (33.3% of 366) patients with focal epilepsy, 37 (31.4% of 118) patients with generalised epilepsy and 4 (25% of 16) patients with unclassifiable epilepsy reported having headache at least 1 day per month. The distribution of headache types in the different epilepsy types is presented in Table 2. The type of epilepsy was not associated with the headache types (*x*^2^ = 2.73, *p* = 0.25).

**Table 2:** Different headache types in patients with focal epilepsy, total numbers (%)

Type of headache	Patients with focal epilepsy	Patients with generalised epilepsy	Patients with unclassifiable epilepsy
MIG	40 (24.5%)	14 (8.6%)	0
with aura	11	2	0
without aura	29	12	0
TTH	45 (27.6%)	9 (5.5%)	0
TAC	5 (3.1%)	0	2 (1.2%)
MIG & TTH	4 (2.4%)	2 (1.2%)	1 (0.6%)
MIG & TAC	5 (3.1%)	2 (1.2%)	0
TTH & TAC	0	0	1 (0.6%)
NCH	23 (14.1%)	10 (6.1%)	0
Total	122 (74.8%)	37 (22.7%)	4 (2.5%)

### AEDs and headache

Of 163 epilepsy patients who reported having headache at least 1 day per month, AEDs were taken by 157 patients (AEDs per patient mean ± SD: 1.3 ± 0.62, [range: 0–4]). Of those patients, 105 (66.9%) received monotherapy, 52 (33.2%) received polytherapy at the time of interview. The distributions of the three main headache types during AED treatment with Topiramate (TPM), Valproic acid (VPA), Lamotrigine (LTG), Carbamazepine (CBZ) and Levetiracetam (LEV) are shown in Table 3.

**Table 3:** Number of patients on mono- and polytherapy reporting the three main headache types MIG, TTH and TAC

AEDs	MIG	TTH	TAC
Topiramate (Monotherapy)	2	0	1
Topiramate (Polytherapy)	3	1	0
Valproic acid (Monotherapy)	5	6	0
Valproic acid (Polytherapy)	7	4	1
Lamotrigine (Monotherapy)	7	7	1
Lamotrigine (Polytherapy)	6	8	1
Carbamazepine (Monotherapy)	8	13	3
Carbamazepine (Polytherapy)	7	5	2
Levetiracetam (Monotherapy)	11	8	1
Levetiracetam (Polytherapy)	14	6	3

Patients who received monotherapy reported less MIG than patients on polytherapy (*x*^2^ = 4.79, *p* = 0.028), whereas there was no difference for TTH and TAC (*p*-values > 0.05).

### Seizure-associated headache

Out of 163 patients of the total study population who had headache, 66 (40.5%) reported seizure-associated headache. Therein, the isolated preictal headache was reported by 8 patients, isolated ictal headache by 2 patients and isolated postictal headache by 30 patients. One patient had preictal and ictal headache, 17 patients had preictal and postictal headache, 3 patients showed ictal and postictal headache and 5 patients had preictal, ictal and postictal headaches.

### Relationship between headache and seizure freedom

In the study population of all epilepsy patients (*n* = 500), 177 (35%) patients were not seizure-free and 163 (32.6%) patients had headache. The association between headache and seizure freedom was marginally significant (*x*^2^ = 3.25, *p* = 0.071). Patients that experienced headache were disproportionally more likely to not achieve seizure freedom.

## Discussion

The present work represents the first systematic approach to assess the headache distribution of the three most common headache types and their associations in patients with epilepsy.

The results indicate that primary headaches, especially MIG and TTH, are common in epilepsy, which is in line with previous studies [9, 25]. About one-third of patients with epilepsy were suffering from some kind of headache at least 1 day per month. MIG and TTH are the most common headache types. Around one-third of the epilepsy patients reported MIG, demonstrating a higher prevalence of MIG compared to the general population (10.0%) [26]. In addition, this is congruent with data about the frequency of MIG in the epilepsy population [9]. Similar results were found in a study in children with juvenile myoclonic epilepsy showing higher migraine frequency in patients compared to a healthy control group [25]. The prevalence of TTH in our study group was even lower than in the general adult population (38.0%) [26] but comparable to findings of TTH in adults and children with epilepsy [9, 25]. The prevalence of TAC in our study was 3.0% higher than in epidemiological studies, including five European studies which have reported a lifetime prevalence of TAC with values ranging from 0.06 to 0.3% [26]. Additionally, we found seven patients with a combination of MIG and TAC as well as one patient with TTH and TAC. We could not determine whether this result is due to a higher prevalence of TAC in patients with epilepsy, because there are no data available concerning this theme to date. TAC is suspected to show a pathophysiology in the trigeminovascular system, autonomic system, hypothalamus, and vagus nerve [27, 28]. The activation of peripheral and central trigeminovascular neurons by seizures may explain the higher amount of TAC in the epilepsy cohort [29].

Regarding the gender effect, headache was more prevalent in female patients (43.2%) compared to males (22.0%). Thereof, 37.0% of female patients matched the criteria for MIG compared to 25% of male patients. In the general population, MIG seems to be more frequent in females with a male-to-female ratio for MIG among adults ranging from 1:2 to 1:3 [26]. Previous epidemiological studies have shown a 4:5 male-to-female ratio for TTH, whereas we found a slightly higher amount of male epilepsy patients reporting TTH compared to females [26]. Further, the prevalence of chronic headache (9.2%) was slightly higher than that reported in the general population (4.8%) [26, 30].

The type of epilepsy did not influence the frequency of headache types. Within the different types of generalised, focal and unclassifiable epilepsy, the headache types showed similar amounts. No patients with generalised epilepsy showed TAC, whereas mostly focal epilepsy patients indicated this rare type of headache.

AEDs are widely used. In addition to the treatment of epilepsy, TPM and VPA are also approved in many European countries for MIG prevention [11, 12, 14–17]. In our study, we focused on the most commonly used AEDs such as TPM, VPA, LEV, CBZ and LTG. Patients on monotherapy with these AEDs reported MIG less frequently compared to patients on polytherapy. This supports previous findings of less adverse effects during monotherapy compared to polytherapy in patients with epilepsy [31, 32]. There was no difference in the incidence of MIG between these AEDs indicating that as well as TPM and VPA being used for MIG prophylaxis, LEV, CBZ and LTG may also help to reduce MIG. However, patients treated with TPM and VPA had the lowest amount of reported MIG. LEV was found to reduce the frequency and intensity of MIG and to be a well-tolerated drug with few side effects in previous studies [33–35]. Regarding the effects of TPM on migraine prophylaxis, we have not been able to evaluate consistent data, because only two epileptic patients with MIG were treated with TPM monotherapy.

Around 40% of the patients with epilepsy reporting headache indicated having seizure-associated headaches expressed by preictal, ictal or postictal headaches. This is consistent with previous studies which reported a prevalence of any seizure-associated headache up to 47% in epileptic patients [36–39]. Postictal headache was the most common seizure-associated headache in our cohort which is in line with previous findings in adult patients with epilepsy [40]. Postictal headache was found to be associated to drug-resistance, generalised epilepsy and family history [40]. Furthermore, there was a weak association between headache and seizure freedom: patients with headache were more likely to not be seizure-free compared to patients without headache.

The main limitation of this study is that headache was assessed only retrospectively at a single time point. Future studies should focus on prospectively assessed headache appearance using a headache diary and on the prevalence of TAC in epilepsy patients as no studies are reporting on this type of headache. However, due to the high number of patients recruited in this study, the results are quite representative for the epilepsy population.

## Conclusion

In patients with epilepsy, MIG and TTH seem to be the most frequent headache types. Headache is more prevalent in female patients compared to male patients, whereas the type of epilepsy did not show any significant impact on the headache type. AED monotherapy reduced the frequency of MIG when compared to polytherapy. The promising results of all AEDs assessed in the study being able to reduce the frequency of MIG suggests that even AEDs that are not yet approved for MIG prophylaxis may be effective in patients with epilepsy.

## Data Availability

The data presented in this study are available on request from the corresponding author.

## References

[1] Keezer MR, Sisodiya SM, Sander JW (2016). Comorbidities of epilepsy: current concepts and future perspectives. Lancet Neurol.

[2] Kim DW, Lee SK (2017). Headache and epilepsy. J Epilepsy Res.

[3] Papetti L, Nicita F, Parisi P, Spalice A, Villa MP, Kasteleijn-Nolst Trenite DG (2013). "Headache and epilepsy"–how are they connected?. Epilepsy Behav.

[4] Headache Classification Subcommittee of the International Headache S. The international classification of headache disorders: 2nd edition. *Cephalalgia* 2004; 24(Suppl 1):9–160. 10.1111/j.1468-2982.2003.00824.x 14979299

[5] Headache Classification Committee of the International Headache S. The international classification of headache disorders, 3rd edition (beta version). *Cephalalgia* 2013; **33**(9):629–808. 10.1177/0333102413485658 23771276

[6] Ottman R, Lipton RB (1994). Comorbidity of migraine and epilepsy. Neurology.

[7] Bigal ME, Lipton RB, Cohen J, Silberstein SD (2003). Epilepsy and migraine. Epilepsy Behav.

[8] Group HS (2010). Multi-center study on migraine and seizure-related headache in patients with epilepsy. Yonsei Med J.

[9] Duko B, Ayalew M, Toma A (2020). The epidemiology of headaches among patients with epilepsy: a systematic review and meta-analysis. J Headache Pain.

[10] Storey JR, Calder CS, Hart DE, Potter DL (2001). Topiramate in migraine prevention: a double-blind, placebo-controlled study. Headache.

[11] Brandes JL, Saper JR, Diamond M, Couch JR, Lewis DW, Schmitt J, Neto W, Schwabe S, Jacobs D, Group M-S (2004). Topiramate for migraine prevention: a randomized controlled trial. JAMA.

[12] Silberstein SD, Ben-Menachem E, Shank RP, Wiegand F (2005). Topiramate monotherapy in epilepsy and migraine prevention. Clin Ther.

[13] Ruiz L, Ferrandi D (2009). Topiramate in migraine progression. J Headache Pain.

[14] Diener HC, Tfelt-Hansen P, Dahlof C, Lainez MJ, Sandrini G, Wang SJ, Neto W, Vijapurkar U, Doyle A, Jacobs D (2004). Topiramate in migraine prophylaxis–results from a placebo-controlled trial with propranolol as an active control. J Neurol.

[15] Sorensen KV (1988). Valproate: a new drug in migraine prophylaxis. Acta Neurol Scand.

[16] Hering R, Kuritzky A (1992). Sodium valproate in the prophylactic treatment of migraine: a double-blind study versus placebo. Cephalalgia.

[17] Freitag FG, Collins SD, Carlson HA, Goldstein J, Saper J, Silberstein S, Mathew N, Winner PK, Deaton R, Sommerville K (2002). A randomized trial of divalproex sodium extended-release tablets in migraine prophylaxis. Neurology.

[18] Lampl C, Marecek S, May A, Bendtsen L (2006). A prospective, open-label, long-term study of the efficacy and tolerability of topiramate in the prophylaxis of chronic tension-type headache. Cephalalgia.

[19] Steiner TJ, Stovner LJ, Katsarava Z, Lainez JM, Lampl C, Lanteri-Minet M, Rastenyte D, Ruiz de la Torre E, Tassorelli C, Barre J (2014). The impact of headache in Europe: principal results of the Eurolight project. J Headache Pain.

[20] Wei DY, Yuan Ong JJ, Goadsby PJ (2018). Cluster headache: epidemiology, pathophysiology, clinical features, and diagnosis. Ann Indian Acad Neurol.

[21] Dalla Volta G, Di Monda V, Bariselli M, Vignolo LA (1992). Headache and epilepsy: a case report of the unusual association of cluster headache and epilepsy. Ital J Neurol Sci.

[22] Scheffer IE, Berkovic S, Capovilla G, Connolly MB, French J, Guilhoto L, Hirsch E, Jain S, Mathern GW, Moshé SL (2017). ILAE classification of the epilepsies: position paper of the ILAE Commission for Classification and Terminology. Epilepsia.

[23] Fritsche G, Hueppe M, Kukava M, Dzagnidze A, Schuerks M, Yoon MS, Diener HC, Katsarava Z (2007). Validation of a german language questionnaire for screening for migraine, tension-type headache, and trigeminal autonomic cephalgias. Headache.

[24] Headache Classification Committee of the International Headache Society (IHS). The international classification of headache disorders, 3rd edition. Cephalalgia 2018; 38(1):1–211. 10.1177/0333102417738202 29368949

[25] Dedei Daryan M, Güveli BT, Baslo SA, Mulhan K, Sarı H, Balçık ZE, Ataklı D (2018). Prevalence and clinical characteristics of headache in juvenile myoclonic epilepsy: experience from a tertiary epilepsy center. Neurol Sci.

[26] Jensen R, Stovner LJ (2008). Epidemiology and comorbidity of headache. Lancet Neurol.

[27] Burish MJ, Rozen TD (2019). Trigeminal autonomic cephalalgias. Neurol Clin.

[28] Benoliel R (2012). Trigeminal autonomic cephalgias. Br J Pain.

[29] Melo-Carrillo A, Schain AJ, Strassman AM, Burstein R (2020). Activation of peripheral and central trigeminovascular neurons by seizure: implications for ictal and postictal headache. J Neurosci.

[30] Stovner LJ, Zwart JA, Hagen K, Terwindt GM, Pascual J (2006). Epidemiology of headache in Europe. Eur J Neurol.

[31] Guberman A (1998). Monotherapy or polytherapy for epilepsy?. Can J Neurol Sci.

[32] Andrew T, Milinis K, Baker G, Wieshmann U (2012). Self reported adverse effects of mono and polytherapy for epilepsy. Seizure.

[33] Miller GS (2004). Efficacy and safety of levetiracetam in pediatric migraine. Headache.

[34] Brighina F, Palermo A, Aloisio A, Francolini M, Giglia G, Fierro B (2006). Levetiracetam in the prophylaxis of migraine with aura: a 6-month open-label study. Clin Neuropharmacol.

[35] Pizza V, Busillo V, Agresta A, Bisogno A, Capasso A (2011). Elderly patients with migraine: an open-label study on prophylaxis therapy with levetiracetam. Cent Nerv Syst Agents Med Chem.

[36] Leniger T, Isbruch K, von den Driesch S, Diener HC, Hufnagel A (2001). Seizure-associated headache in epilepsy. Epilepsia.

[37] Forderreuther S, Henkel A, Noachtar S, Straube A (2002). Headache associated with epileptic seizures: epidemiology and clinical characteristics. Headache.

[38] Ito M, Adachi N, Nakamura F, Koyama T, Okamura T, Kato M, Kanemoto K, Nakano T, Matsuura M, Hara S (2004). Characteristics of postictal headache in patients with partial epilepsy. Cephalalgia.

[39] Yankovsky AE, Andermann F, Bernasconi A (2005). Characteristics of headache associated with intractable partial epilepsy. Epilepsia.

[40] Caprara F, Letícia A, Rissardo JP, Leite MTB, Silveira JOF, Jauris PGM, Arend J, Kegler A, Royes F, Fernando L (2020). Characteristics of post-ictal headaches in patients with epilepsy: a longitudinal study. Seizure.

